# Genetic influences on right ventricular systolic pressure (RVSP) in chronic obstructive pulmonary disease (COPD)

**DOI:** 10.1186/1471-2466-12-25

**Published:** 2012-06-13

**Authors:** Janet G Shaw, Annette G Dent, Linda H Passmore, Darryl J Burstow, Rayleen V Bowman, Paul V Zimmerman, Kwun M Fong, Ian A Yang

**Affiliations:** 1 Department of Thoracic Medicine, The Prince Charles Hospital, Rode Rd, Chermside, Brisbane, QLD 4032, Australia; 2 Department of Echocardiography, The Prince Charles Hospital, Rode Rd, Chermside, Brisbane, QLD, 4032, Australia; 3 School of Medicine, The University of Queensland, St Lucia, Brisbane, QLD, 4067, Australia

**Keywords:** COPD, Pulmonary hypertension, Genetic polymorphism

## Abstract

**Background:**

Pulmonary hypertension (PH) is a complication of chronic obstructive pulmonary disease (COPD). This study examined genetic variations in mediators of vascular remodelling and their association with PH in patients with COPD. In patients with COPD, we genotyped 7 SNPs in 6 candidate PH genes (*NOS3*, *ACE*, *EDN1*, *PTGIS*, *SLC6A4*, *VEGFA*). We tested for association with right ventricular systolic pressure (RVSP), spirometry and gas transfer, and hypoxemia.

****Methods**:**

In patients with COPD, we genotyped 7 SNPs in 6 candidate PH genes (*NOS3*, *ACE*, *EDN1*, *PTGIS*, *SLC6A4*, *VEGFA*). We tested for association with right ventricular systolic pressure (RVSP), spirometry and gas transfer, and hypoxemia.

**Results:**

580 COPD patients were recruited, 341 patients had a transthoracic echocardiogram, with RVSP measurable in 278 patients (mean age 69 years, mean FEV_1_ 50% predicted, mean RVSP 44 mmHg, median history of 50 pack-years). Of the 7 tested SNPs, the *NOS3*-VNTR polymorphism was significantly associated with RVSP in a dose-dependent fashion for the risk allele: mean RVSP for a/a and a/b genotypes were 52.0 and 46.6 mmHg respectively, compared to 43.2 mmHg for b/b genotypes (*P* = 0.032). No associations were found between RVSP and other polymorphisms. *ACE* II or ID genotypes were associated with a lower FEV_1_% predicted than the *ACE* DD genotype (*P* = 0.028). The *NOS3*-298 TT genotype was associated with lower KCO % predicted than the *NOS3*-298 GG or GT genotype (*P* = 0.031).

**Conclusions:**

The *NOS3*-VNTR polymorphism was associated with RVSP in patients with COPD, supporting its involvement in the pathogenesis of PH in COPD. *ACE* and *NOS3* genotypes were associated with COPD disease severity, but not with the presence of PH. Further study of these genes could lead to the development of prognostic and screening tools for PH in COPD.

## Background

Pulmonary hypertension (PH) is a serious complication of chronic obstructive pulmonary disease (COPD) and develops in 30% to 70% of patients with COPD, increasing their morbidity and mortality [1]. PH is progressive in COPD, with mean pulmonary arterial pressure increasing over time [2,3]. Understanding variations in susceptibility to PH in patients with COPD could significantly enhance diagnosis, risk stratification and therapy for these patients.

Vascular remodelling is the main pathological feature in PH and is mediated via vasoactive molecules [4]. Genes encoding these mediators contain genetic polymorphisms that potentially affect their function and could influence PH in COPD [5-18]. Polymorphisms exist in vasodilator (nitric oxide synthase (*NOS3*) [8,13], prostacyclin synthase (*PTGIS*) [19]) and vasoconstrictor (angiotensin converting enzyme (*ACE*) [17], endothelin-1 (*ET1*), serotonin transporter (*SLC6A4)*[14]) and vascular endothelial growth factor (VEGFA) [20] genes.

We hypothesised that genetic variation in genes encoding mediators acting on pulmonary vessels alters right ventricular systolic pressure (RVSP) in patients with COPD, even after adjustment for clinical factors associated with elevated RVSP. Additionally, we hypothesised that these polymorphisms are associated with COPD disease severity. We selected variants previously associated with vascular disease, in vasoactive mediators of biological importance in pulmonary hypertension.

## Methods

### Participants

Patients with a thoracic physician’s diagnosis of COPD, based on chronic airflow limitation not fully reversible with bronchodilator [21], reduced gas transfer (KCO) and/or emphysema on CT scan, were recruited from thoracic clinics and wards of The Prince Charles Hospital (TPCH). Patients with other major respiratory diseases, including interstitial lung disease, lung cancer, pneumonia, cystic fibrosis, bronchiectasis, pleural effusion and lung surgery, were excluded. All participants gave written informed consent. This study was approved by the research ethics committees of TPCH (EC9865 and EC2108) and The University of Queensland (H372).

### Clinical phenotyping

Participants completed a clinical questionnaire about their lung disease, symptoms, smoking history and ethnicity. One pack-year of smoking was defined as the equivalent of 20 cigarettes smoked per day for one year. Chronic bronchitis was defined as a cough with sputum production for at least 3 months in each of two consecutive years [21].

### Lung function tests

Participants had lung function tests at recruitment, during their routine care at the hospital, whilst clinically stable. Lung function testing consisted of spirometry and single breath carbon monoxide diffusing capacity (DLCO) and performed according to American Thoracic Society/European Respiratory Society Taskforce (ATS/ERS) standards [22-24]. Measurements were compared to predicted values [25,26]. The lung function parameters FEV_1_, slow vital capacity (VC), FEV_1_/VC ratio and KCO were used to characterise COPD.

### Echocardiography

A subgroup of patients had a transthoracic echocardiogram performed as a routine part of their clinical care, using standard techniques [27]. RVSP was calculated using the simplified Bernoulli equation. The pressure difference between the right ventricle and right atrium during systole is reflected by the velocity of the tricuspid regurgitation (TR) signal. RVSP can be estimated from right atrial pressure using the equation RVSP = 4(V_TR_)^2^ + RAP, where V_TR_ is peak TR velocity (m/s) and RAP is mean right atrial pressure (mmHg). The mean RAP is estimated using inferior vena cava (IVC) size and reactivity as per American Society of Echocardiography (ASE) recommendations [28].

### Arterial blood gases

Arterial blood gas (ABG) results were obtained from the patients’ medical records. Arterial blood was drawn from the radial artery while the patients breathed room air.

### Genotyping

Genomic DNA was extracted from peripheral blood using a modified salt extraction method [29]. Polymerase chain reaction (PCR) was used to genotype two polymorphisms, PCR with restriction fragment length polymorphism (RFLP) was used for three polymorphisms, and single base pair extension was used for one polymorphism (see Additional file 1: Table S1). PCR reactions were performed in a final volume of 20 μl containing 10 ng genomic DNA, 0.2 mM of dNTPs, and 0.25 μM of each of the forward and reverse primers. For five polymorphisms, 0.5 U REDTaq DNA polymerase (Sigma, Saint Louis, Missouri, USA) and 10x REDTaq PCR reaction buffer were used. For the SLC6A4 polymorphism, 1.5 U HotStarTaq DNA polymerase (QIAGEN, Hilden, Germany) and 10x HotStar PCR reaction buffer were used, together with 5x Q-Solution. The PCR amplification was performed in a thermal cycler. Genotypes were determined by agarose gel electrophoresis of PCR products. Ethidium bromide was added to each gel at 1 μg/ml final concentration. The stained bands were visualised by the Molecular Imager FX (Bio-Rad, Hercules, California, USA). To ensure reproducibility, 10% of the samples were chosen randomly and repeated for each polymorphism; these genotypes were confirmed by this repeated testing.

The *EDN1* gene polymorphism was genotyped by single base extension with alleles identified by direct mass measurement of the analyte using the SEQUENOM^TM^ MassARRAY® system (MALDI-TOF mass spectrometry) at the Australian Genome Research Facility.

### Statistical methods

SPSS (Statistical Package for the Social Sciences) for Windows Version 17.0 (SPSS Inc, Chicago Illinois, USA) was used. Hardy-Weinberg equilibrium was calculated using the χ^2^ test to compare expected *vs* observed genotype frequencies. Associations of genotypes with RVSP or lung function were performed using ANOVA for additive genotype models (AA *vs* AB *vs* BB), and *t*-tests for dominant (AA *vs* AB + BB) and recessive (AA + AB vs BB) genotype models. Adjustment for clinical confounders was performed using multiple linear regression, in which genotypes and clinical covariates (age, gender, BMI, PaO_2_, FEV_1_% predicted, KCO % predicted) were independent variables, and RVSP was the dependent variable. Analyses were done for all available participants, and also with the subgroup of COPD patients with FEV_1_/VC < 0.7. In all analyses, a two-tailed *P* value of <0.05 was considered significant.

For the *t*-test analyses of RVSP using dominant or recessive models, power calculations indicated that a total of 190 participants with RVSP measurements were required to detect a difference of 15% in mean RVSP in the cohort, with 80% power (based on mean RVSP of 44 mmHg, SD 13 mmHg, *P* value of 0.05, and ratio of genotype groups of at least 1:2, which was the case for all variants studied, except the *NOS3* VNTR which had a lower ratio) (PS Power calculation program [30]). Power varied according to allele frequency and type of genetic analysis.

## Results

### Participant characteristics

580 patients with COPD were studied (Table 1); most were Caucasian males (only two of Asian ethnicity), with a mean age of 68 years. Chronic bronchitis was present in 35% of patients. All were current or former smokers, with the exception of six never smokers who nevertheless satisfied criteria for COPD. 514 patients (89%) had moderate to severe airflow limitation (FEV1% predicted < 80%), with mean FEV_1_ 50% predicted. Gas transfer (KCO) was measured in 97% (561/580) of patients, and was below the lower limit of normal (75% predicted) in 78% (437/561). Mean gas transfer (KCO % predicted) was 58%. ABG results were obtained for 271 patients who also had a measureable RVSP, 81% of the patients had an ABG within 12 months of their echocardiogram.

**Table 1:** Demographic characteristics of participants

**Characteristics**	**All COPD patients**	**Subgroup with RVSP results**	**Subgroup without measureable RVSP**	***P*value**
Males/Females	364/216	167/111	42/21	
%Males/%Females	63/37	60/40	67/33	
Mean (SD) age at recruitment (yr)	68.1 (9.3)	69.3 (9.0)	67.3 (9.8)	0.11
Range	35.1–87.0	38.3-87.0	35.1-81.2	
Median smoking history (pack years)	50	50	47	0.93
(IQR)	(33.8-69.0)	(31.5-69.0)	(34.0-68.8)	
Range	0.0-186.0	0.0-186.0	8.5-172.5	
Chronic bronchitis present (% of total)	201 (35%)	99 (36%)	26 (41%)	0.33
Mean (SD) FEV_1_ (L)	1.2 (0.63)	1.20 (0.55)	1.13 (0.50)	0.40
Range	0.2-4.7	0.2-3.9	0.34-3.09	
Mean (SD) FEV_1_% predicted	50 (21.8)	50 (20.8)	44.5 (18.1)	0.062
Range	7-129	7-129	11.4-89.3	
Mean (SD) VC (L)	2.8 (0.92)	2.7 (0.83)	2.81 (0.87)	0.28
Range	0.9-6.4	0.9-5.6	1.15-4.88	
Mean (SD) VC (L) % predicted	77.3 (17.8)	76.0 (17.4)	75.5 (16.6)	0.88
Range	28.5-132.3	28.8-132.3	36.3-111.0	
Mean (SD) FEV_1_/VC ratio %	45 (15)	45 (14)	40.9 (13.5)	**0.026**
Range	7-80	7-80	17.5-67.9	
Mean (SD) KCO (ml/min/mmHg/L)	2.5 (0.96)	2.4 (0.97)	2.56 (0.97)	0.36
Range	0.44-6.6	0.44-6.6	0.49-4.7	
Mean (SD) KCO % predicted	58 (22.5)	58 (23.4)	59.8 (22.4)	0.60
Range	9.8-134.5	12.6-134.5	9.8-109.8	
Mean (SD) RVSP (mmHg)	44.2 (12.7)	44.2 (12.7)	Unable to	
Range	16.0-94.0	16.0-94.0	measure	
Mean (SD) PaO_2_ (mmHg)	70.5 (11.6)	69.4 (11.8)	71.7 (11.5)	0.23
Range	35.0-99.0	35.0-99.0	53.0-95.0	
Mean (SD) PaCO_2_ (mmHg)	41.3 (7.7)	41.6 (8.1)	40.8 (7.7)	0.49
Range	26.0-76.0	27.0-76.0	26.0-62.0	
Mean (SD) BMI (kg/m^2^)	25.0 (5.5)	24.9 (5.3)	26.0 (5.5)	0.14
Range	13.0-46.1	13.5-44.0	15.9-46.1	

### Echocardiography

Echocardiography was performed in 341 of the 580 (59%) patients. RVSP was measurable in 278 of the 341 patients who had an echocardiogram (82% of total 341). The demographic characteristics of patients who had echocardiography did not differ significantly from those of patients with no echocardiography. Echocardiography was performed but no RVSP measurement was possible in 63 patients either because there was no tricuspid regurgitation or the echocardiogram was technically difficult and echo signals could not be obtained. Demographic and disease characteristics were similar between patients with and without measurable RVSP, except for a lower FEV_1_/VC ratio in those without measurable RVSP. Both these subgroups had similar characteristics to the whole cohort (Table 1). RVSP measurements ranged from 16 to 94 mmHg, 115 patients were within the normal range chosen for this study (15-39 mmHg) and 163 patients (59%) had RVSP ≥ 40 mmHg [31].

Data on the left ventricular ejection fraction (EF), mitral valve and aortic valve were collected for each patient. 13 patients had missing EF measurements and 6 patients had missing information on both mitral and aortic valves. A normal EF (>50%) was recorded for 275 (80% of 328) of the patients (range 11% - 82%, mean (SD) 59% (12.2)). 60% (205 of 335) of the patients had a normal mitral valve, with 123 (36%) having some degree of mitral regurgitation (of whom 38 had moderate to severe mitral regurgitation), 5 (1.5%) having mitral stenosis (of whom 3 had moderate to severe mitral stenosis) and 2 (0.6%) having both. A normal aortic valve was found in 48% (165 of 335) of the patients, 103 (30%) had aortic valve sclerosis, 54 (16%) had aortic regurgitation (of whom 14 had moderate to severe aortic regurgitation) and 13 (3.8%) had aortic stenosis (of whom 7 had moderate to severe aortic stenosis).

### Genotypes

98.6% to 100% of all the patients were successfully genotyped by the PCR or PCR-RFLP methods, and the *EDN1* polymorphism had a 95.7% success rate using the single base extension method. The distribution of the genotypes for 6 of the 7 polymorphisms were in Hardy-Weinberg equilibrium (HWE), with the exception being *NOS3*-Glu298Asp SNP which deviated from HWE (*P* = 0.02).

### Association of genotypes with RVSP measurements

One-way ANOVA was used to test the relationship between genotype and RVSP (Table 2). There was a significant association between RVSP and *NOS3*-VNTR SNP genotypes (*P* = 0.032), with highest mean RVSP in patients with 4aa genotype of the VNTR. There were no statistically significant associations with RVSP for SNP genotypes of other genes tested (*VEGFA*, *ACE*, *SLC6A4*, *PTGIS*, *NOS3*-298 and *EDN1*). The distribution of genotypes in the subgroup of COPD patients with a RVSP measurement were in agreement with the HWE predicted frequencies except for *EDN1* (*P* = 0.04).

**Table 2:** Association of genotypes with RVSP measurements in COPD patients with an RVSP measurement, using ANOVA

**Gene**	**Genotypes**	**Number**	**Mean RVSP (mmHg)**	**Standard** **Deviation**	***P*value**
*VEGFA*	GG	123	42.8	12.0	0.24
	GC	117	45.2	13.6	
	CC	38	46.0	12.1	
		Total 278	44.2	12.7	
*NOS3*-VNTR	4bb	199	43.2	11.8	**0.032**
	4ab	70	46.6	13.8	
	4aa	8	52.0	20.2	
		Total 277	44.3	12.7	
*ACE*	II	69	45.6	14.2	0.61
	ID	139	44.0	12.5	
	DD	69	43.6	11.6	
		Total 277	44.3	12.7	
*SLC6A4*	LL	90	42.7	12.6	0.43
	LS	131	44.8	13.2	
	SS	53	45.1	11.3	
		Total 274	44.2	12.6	
*PTGIS*	CC	172	43.9	12.2	0.83
	CA	90	45.0	13.6	
	AA	15	44.3	13.8	
		Total 277	44.3	12.7	
*NOS3*-298	GG	120	44.4	12.6	0.83
	GT	135	43.9	12.4	
	TT	22	45.6	15.7	
		Total 277	44.3	12.7	
*EDN1*	GG	159	43.5	12.8	0.15
	GT	83	46.0	12.8	
	TT	24	40.7	9.3	
		Total 266	44.0	12.6	

Some samples could not be genotyped, making the totals less than 278.

When considering only those COPD patients with FEV1/VC < 0.7 (n = 263), the *NOS3*-VNTR SNP genotype association remained statistically significant in a *t*-test analysis (4bb, mean (SD) 43.5 mmHg (11.8), n = 186 vs 4ab/4aa, mean (SD) 47.1 mmHg (14.6) n = 77, *P* = 0.034).

### Association of clinical factors with RVSP measurements

Clinical factors potentially confounding the association of genotypes with RVSP include arterial PaO_2_, FEV_1_% predicted, KCO % predicted and FEV_1_/VC ratio, all of which showed weak inverse correlations with RVSP (Table 3).

**Table 3:** Correlation of clinical factors with RVSP in COPD patients with an RVSP measurement

**Clinical Factor**	**Number**	**Correlation (r)**	***P*value**
PaO_2_ vs RVSP	222	−0.245	**0.00023**
PaCO_2_ vs RVSP	222	0.041	0.55
FEV_1_% predicted vs RVSP	278	−0.118	**0.050**
KCO percent predicted vs RVSP	268	−0.258	**0.000019**
Age at recruitment vs RVSP	278	0.117	0.050
Smoking history: pack years vs RVSP	278	−0.042	0.48
FEV_1_/VC ratio vs RVSP	278	−0.133	**0.027**
BMI vs RVSP	278	−0.115	0.055

### Association of genotypes with lung function impairment

To detect disease-modifying effects of the candidate SNPs one-way ANOVA analyses were performed between genotypes and FEV_1_ (% predicted) and KCO (% predicted). No significant associations were found (Tables 4 and 5).

**Table 4:** **Association of genotype groups with FEV**_**1**_**% predicted, using ANOVA**

**Gene**	**Genotypes**	**Number**	**Mean FEV**_**1**_**% predicted**	**Standard** **Deviation**	***P*value**
*VEGFA*	GG	246	48.7	21.0	0.23
	GC	256	50.6	22.7	
	CC	75	53.5	20.4	
		Total 577		21.7	
*NOS3*-VNTR	4bb	414	50.0	22.3	0.80
	4ab	148	51.0	20.6	
	4aa	15	47.7	17.7	
		Total 577		21.7	
*ACE*	II	142	49.6	20.9	0.09
	ID	283	48.8	21.9	
	DD	153	53.6	22.2	
		Total 578		21.8	
*SLC6A4*	LL	179	49.7	22.7	0.97
	LS	285	50.2	21.2	
	SS	107	50.2	22.1	
		Total 571		21.8	
*PTGIS*	CC	336	50.0	21.0	0.77
	CA	200	49.9	22.8	
	AA	42	52.4	23.1	
		Total 578		21.7	
*NOS3*-298	GG	258	50.1	21.0	0.61
	GT	276	50.9	22.8	
	TT	41	47.4	18.3	
		Total 575		21.7	
*EDN1*	GG	322	50.0	22.4	0.77
	GT	178	50.6	21.3	
	TT	42	47.9	19.1	
		Total 542		21.8	

Some samples could not be genotyped, making the totals less than 580.

**Table 5:** Association of genotype groups with KCO % predicted, in COPD patients with a KCO measurement, using ANOVA

**Gene**	**Genotypes**	**Number**	**Mean KCO % predicted**	**Standard Deviation**	***P*value**
*VEGFA*	GG	241	58.4	23.0	0.88
	GC	244	57.7	21.8	
	CC	73	59.1	23.9	
		Total 558		22.6	
*NOS3*-VNTR	4bb	401	58.1	22.9	0.36
	4ab	142	59.1	21.5	
	4aa	15	50.3	24.5	
		Total 558		22.6	
*ACE*	II	136	60.3	24.9	0.42
	ID	273	57.3	22.1	
	DD	150	57.6	21.2	
		Total 559		22.6	
*SLC6A4*	LL	176	58.0	22.4	0.84
	LS	275	58.7	22.8	
	SS	101	57.2	22.0	
		Total 552		22.5	
*PTGIS*	CC	327	58.5	21.4	0.11
	CA	192	56.2	23.8	
	AA	40	64.3	25.0	
		Total 559		22.6	
*NOS3*-298	GG	250	58.5	24.0	0.10
	GT	265	58.9	21.7	
	TT	41	50.9	16.7	
		Total 556			
*EDN1*	GG	310	57.5	22.4	0.17
	GT	173	57.5	22.7	
	TT	40	64.6	24.5	
		Total 523		22.7	

Some samples could not be genotyped, making the totals less than 580.

### Linear regression modelling of associations with RVSP measurements

Multiple linear regression modelling was used to test whether genotypes associated with RVSP remained significantly associated, when controlled for clinical factors. Arterial PaO_2_ and KCO % predicted were significantly associated with RVSP in all analyses (see Additional file 1: Table S2). The association with *NOS3*-VNTR, as grouped genotypes 4aa + 4ab *vs* 4bb, remained significantly associated with RVSP, even when controlling for the clinical factors (see Additional file 1: Table S3).

### Additional genetic modelling

Patients with the *NOS3*-VNTR 4aa or 4ab genotypes had significantly higher RVSP levels than the *NOS3*-VNTR 4bb genotype (see Additional file 1: Table S3). There were no other significant differences observed between genotypes and RVSP when using dominant and recessive genetic models. The *ACE* II or ID genotypes were associated with a significantly lower FEV_1_% predicted than the DD genotype (*P* = 0.028) (see Additional file 1: Table S4). All other genotypes were not associated with FEV_1_% predicted. The *NOS3*-298 TT genotype was associated with lower KCO % predicted than GG or GT genotypes (*P* = 0.031) (see Additional file 1: Table S5). No other significant differences were found between genotypes and KCO % predicted.

## Discussion

Few previous studies have examined multiple polymorphisms in relation to PH associated with COPD. In our study we examined seven SNPs in six candidate genes which encode mediators that act on pulmonary vessels. We tested whether these SNPs were associated with RVSP, which is a measure of PH. We found that patients with the *NOS3*-VNTR 4aa or 4ab genotype had significantly higher RVSPs than those with the *NOS3*-VNTR 4bb genotype. In contrast, a study of 42 COPD patients and 40 controls found that the *NOS3*-VNTR 4bb genotype was associated with PH in COPD [13]. The smaller number of patients in their study raises the possibility of a type I error Further studies are required to validate our findings.

There have been conflicting functional studies of the *NOS3*-VNTR 4aa genotype and plasma nitrite and nitrate (NOx) levels. A study of 428 healthy Caucasian members of 108 nuclear families found significantly higher levels of plasma NOx associated with the 4aa genotype compared to the 4bb and 4ab genotypes [32]. However another study found that there was a strong association between plasma NOx levels and the *NOS3*-VNTR polymorphism in 413 healthy Japanese subjects, the subjects with the 4aa genotype having significantly lower NOx levels [33]. The discordant results between these two studies may be due to ethnicity or methodological differences in measuring NOx levels. The functional results from this second study would support our finding of the 4aa genotype (with potentially lower nitric oxide levels and therefore less vasodilatation) being associated with higher RVSP (higher vascular resistance). The exact functional mechanisms of how the VNTR, or a nearby SNP which is in linkage disequilibrium with it, affects either nitric oxide or vascular remodelling, needs further elucidation.

We identified a number of clinical factors as being significantly associated with elevated RVSP on univariate analysis. These factors are well-known clinical markers of severity of COPD, and would be expected to correlate with elevated RVSP, since PH is related to COPD severity [31]. The *NOS3*-VNTR polymorphism was significantly associated with RVSP, and remained so when controlling for these factors.

Considering potential links between COPD severity and PH, we examined the effect of SNPs in relation to respiratory function tests. Patients with the *ACE* II or ID genotypes showed a statistically significantly lower FEV_1_% predicted, albeit a clinically small difference, than the *ACE* DD genotypes. Analysis of the other genetic models for *ACE* genotypes did not show associations. This is in contrast to a previous study of Caucasian Mediterraneans which found a higher frequency of *ACE* DD genotypes in 74 male smokers with COPD than in 77 male smokers with normal lung function (odds ratio 2.2) [34]. Additional studies are required to clarify this relationship.

Patients with the *NOS3*-298 TT genotype had significantly lower KCO than those with the GG or GT genotypes. Sun and co-workers’ hypothesis [8] supports our results in that the lower NO levels associated with the TT genotype would potentially predispose those patients to greater lung tissue damage from cigarette smoke reflected in a reduced KCO.

Potential limitations of this study should be considered. Two of the SNPs showed minor deviation in Hardy-Weinberg equilibrium, although these did not show positive associations. The reason for the minor deviation could include chance or differences in population sampling; we had performed repeat genotyping in 10% of samples and the results were concordant. In some COPD patients, RVSP measurement was not successful because of hyperinflated lungs causing a large retrosternal window, or because the tricuspid regurgitant jet was insufficient to enable calculation of RVSP. Use of echo contrast may have increased the yield of RVSP measurements in these cases. Given the exploratory nature of this study of 7 SNPs and RVSP in COPD, we did not correct for multiple comparisons, and the statistical significance of the results should be considered in light of this. Even with this relatively large cohort of patients, replication in other cohorts is needed. Functional analysis of polymorphisms in model systems and genome-wide association studies of PH in COPD would be worthwhile in the future.

## Conclusions

This study has shown a significant association between RVSP in COPD patients and the *NOS3*-VNTR 4aa or 4ab genotype. We also found associations between the *ACE* II or ID genotypes and lower FEV_1_% predicted and the *NOS3*-298 TT genotype and lower KCO % predicted. These results suggest that these polymorphisms may influence disease phenotype in COPD patients. Further study of these genes could lead to the development of prognostic and screening tools for PH in COPD, eventually leading to novel therapy targeting these pathways.

## Competing interests

The authors declare they have no competing interests.

## Authors’ contributions

IAY, JGS, RVB, PVZ and KMF designed the study and analysed data. JGS carried out the genotyping. JGS and LHP recruited and phenotyped patients. JGS and AGD carried out and interpreted lung function testing. DJB was responsible for the echocardiographs. JGS and IAY carried out the statistical analyses and interpretation of the data. JGS and IAY drafted the manuscript, and all authors contributed to and approved the manuscript.

## Pre-publication history

The pre-publication history for this paper can be accessed here:

http://www.biomedcentral.com/1471-2466/12/25/prepub

## Supplementary Material

Additional file 1 Table **S1: Candidate genes and polymorphisms.** Table **S2**: Multiple regression analysis for RVSP. Table **S3**: Association of genotypes with RVSP measurements, using *t*-test. Table **S4**: Association of genotypes with FEV1% predicted, using *t*-test. Table **S5**: Association of genotypes vs KCO percent predicted, using *t*-test. Click here for file
