# Supplementary material for: Genetic influences on right ventricular systolic pressure (RVSP) in chronic obstructive pulmonary disease (COPD)
Source: BMC Pulm Med. 2012 Jun 13;12:25. doi: 10.1186/1471-2466-12-25 (PMC3431274; doi:10.1186/1471-2466-12-25)
Supplement: Additional file 1 — Table S1: Candidate genes and polymorphisms. Table S2: Multiple regression analysis for RVSP. Table S3: Association of genotypes with RVSP measurements, using t-test. Table S4: Association of genotypes with FEV1% predicted, using t-test. Table S5: Association of genotypes vs KCO percent predicted, using t-test. [file 1471-2466-12-25-S1.doc]

S1: Candidate genes and polymorphisms

| **Gene name** | **Gene symbol** | **Chromosome** | **Polymorphism** | **Reference SNP ID** | **Genotyping method** |
| --- | --- | --- | --- | --- | --- |
| Angiotensin converting enzyme (peptidyl-dipeptidase A)1 | *ACE* | 17q23.3 | Intron 16:  287bp insertion/deletion (I/D) | rs4646994 | PCR |
| Endothelin 1 | *EDN1* | 6p24.1 | Exon 5:  Lys198Asn  (594G>T) | rs5370 | SEQUENOM^TM^ MassARRAY® (Australian Genome Research Facility) |
| Nitric oxide synthase 3 (endothelial cell) | *NOS3* | 7q36 | Intron 4:  27bp VNTR | in/del  (no rsID) | PCR |
|  |  |  | Exon 7:  Glu298Asp  (894G>T) | rs1799983 | PCR-RFLP |
| Prostaglandin I2 (prostacyclin) synthase | *PTGIS* | 20q13.13 | Exon 8:  Codon 373, synonymous  (1117C>A) | rs5629 | PCR-RFLP |
| Solute carrier family 6 (neurotransmitter transporter, serotonin), member 4 | *SLC6A4* | 17q11.1-q12 | Promoter:  Repeats:  Long/short (L/S) | in/del  (no rsID) | PCR-RFLP |
| Vascular endothelial growth factor A | *VEGFA* | 6p21.3 | 5’-untranslated region  +405G>C (also known as –634G>C) | rs2010963 | PCR-RFLP |

S2: Multiple regression analysis for RVSP

Tables show combinations of explanatory variables, including clinical and genetic factors, where available.

| **RVSP vs Predictors** | **Standardised Coefficients** | ***P* value** |
| --- | --- | --- |
| PaO_2_ | -0.245 | **0.00023** |

| **RVSP vs Predictors** | **Standardised Coefficients** | ***P* value** |
| --- | --- | --- |
| PaO_2_ | -0.196 | **0.005** |
| FEV_1_ percent predicted | 0.028 | 0.69 |
| KCO percent predicted | -0.243 | **0.001** |
| Age at recruitment | 0.098 | 0.163 |
| BMI | -0.036 | 0.62 |

| **RVSP vs Predictors** | **Standardised Coefficients** | ***P* value** |
| --- | --- | --- |
| PaO_2_ | -0.201 | **0.004** |
| FEV_1_ percent predicted | 0.096 | 0.42 |
| KCO percent predicted | -0.231 | **0.003** |
| Age at recruitment | 0.092 | 0.19 |
| BMI | -0.038 | 0.61 |
| FEV_1_/VC ratio | -0.084 | 0.48 |

| **RVSP vs Predictors** | **Standardised Coefficients** | ***P* value** |
| --- | --- | --- |
| PaO_2_ | -0.201 | **0.002** |
| FEV_1_ percent predicted | 0.040 | 0.57 |
| KCO percent predicted | -0.229 | **0.002** |
| Age at recruitment | 0.086 | 0.21 |
| BMI | -0.046 | 0.52 |
| *NOS3*-VNTR (4aa +4ab) or 4bb | -0.195 | **0.003** |

| **RVSP vs Predictors** | **Standardised Coefficients** | ***P* value** |
| --- | --- | --- |
| PaO_2_ | -0.190 | **0.006** |
| FEV_1_ percent predicted | 0.015 | 0.83 |
| KCO percent predicted | -0.239 | **0.002** |
| Age at recruitment | 0.097 | 0.16 |
| BMI | -0.042 | 0.56 |
| *VEGFA* (GG + GC) or CC | 0.112 | 0.09 |

| **RVSP vs Predictors** | **Standardised Coefficients** | ***P* value** |
| --- | --- | --- |
| PaO_2_ | -0.195 | **0.005** |
| FEV_1_ percent predicted | 0.024 | 0.73 |
| KCO percent predicted | -0.243 | **0.001** |
| Age at recruitment | 0.095 | 0.18 |
| BMI | -0.035 | 0.63 |
| *VEGFA* GG or (GC+CC) | 0.062 | 0.34 |

| **RVSP vs Predictors** | **Standardised Coefficients** | ***P* value** |
| --- | --- | --- |
| PaO_2_ | -0.190 | **0.007** |
| FEV_1_ percent predicted | 0.031 | 0.66 |
| KCO percent predicted | -0.245 | **0.001** |
| Age at recruitment | 0.100 | 0.16 |
| BMI | -0.040 | 0.59 |
| ACE (II + ID) or DD | -0.028 | 0.67 |

| **RVSP vs Predictors** | **Standardised Coefficients** | ***P* value** |
| --- | --- | --- |
| PaO_2_ | -0.189 | **0.007** |
| FEV_1_ percent predicted | 0.034 | 0.64 |
| KCO percent predicted | -0.246 | **0.001** |
| Age at recruitment | 0.096 | 0.17 |
| BMI | -0.036 | 0.63 |
| *ACE* II or (ID + DD) | -0.063 | 0.33 |

| **RVSP vs Predictors** | **Standardised Coefficients** | ***P* value** |
| --- | --- | --- |
| PaO_2_ | -0.194 | **0.005** |
| FEV_1_ percent predicted | 0.024 | 0.74 |
| KCO percent predicted | -0.247 | **0.001** |
| Age at recruitment | 0.099 | 0.16 |
| BMI | -0.035 | 0.63 |
| *NOS3*-298 (GG + GT) or TT | -0.032 | 0.63 |

| **RVSP vs Predictors** | **Standardised Coefficients** | ***P* value** |
| --- | --- | --- |
| PaO_2_ | -0.196 | **0.005** |
| FEV_1_ percent predicted | 0.028 | 0.69 |
| KCO percent predicted | -0.243 | **0.001** |
| Age at recruitment | 0.097 | 0.17 |
| BMI | -0.036 | 0.62 |
| *NOS3*-298 GG or (GT + TT) | 0.003 | 0.96 |

| **RVSP vs Predictors** | **Standardised Coefficients** | ***P* value** |
| --- | --- | --- |
| PaO_2_ | -0.188 | **0.009** |
| FEV_1_ percent predicted | 0.032 | 0.66 |
| KCO percent predicted | -0.229 | **0.003** |
| Age at recruitment | 0.092 | 0.19 |
| BMI | -0.026 | 0.73 |
| *SLC6A4* (LL + LS) or SS | 0.037 | 0.58 |

| **RVSP vs Predictors** | **Standardised Coefficients** | ***P* value** |
| --- | --- | --- |
| PaO_2_ | -0.182 | **0.010** |
| FEV_1_ percent predicted | 0.031 | 0.66 |
| KCO percent predicted | -0.235 | **0.002** |
| Age at recruitment | 0.088 | 0.21 |
| BMI | -0.021 | 0.77 |
| *SLC6A4* LL or(LS + SS) | 0.078 | 0.24 |

| **RVSP vs Predictors** | **Standardised Coefficients** | ***P* value** |
| --- | --- | --- |
| PaO_2_ | -0.190 | **0.006** |
| FEV_1_ percent predicted | 0.033 | 0.64 |
| KCO percent predicted | -0.243 | **0.001** |
| Age at recruitment | 0.097 | 0.17 |
| BMI | -0.036 | 0.62 |
| *PTGIS* (CC + CA) or (AA) | -0.046 | 0.48 |

| **RVSP vs Predictors** | **Standardised Coefficients** | ***P* value** |
| --- | --- | --- |
| PaO_2_ | -0.190 | **0.006** |
| FEV_1_ percent predicted | 0.030 | 0.68 |
| KCO percent predicted | -0.245 | **0.001** |
| Age at recruitment | 0.100 | 0.16 |
| BMI | -0.037 | 0.61 |
| *PTGIS* CC or (CA + AA) | -0.012 | 0.85 |

| **RVSP vs Predictors** | **Standardised Coefficients** | ***P* value** |
| --- | --- | --- |
| PaO_2_ | -0.209 | **0.004** |
| FEV_1_ percent predicted | -0.003 | 0.97 |
| KCO percent predicted | -0.216 | **0.006** |
| Age at recruitment | 0.066 | 0.36 |
| BMI | -0.029 | 0.70 |
| *EDN1* (GG + GT) or TT | -0.046 | 0.50 |

| **RVSP vs Predictors** | **Standardised Coefficients** | ***P* value** |
| --- | --- | --- |
| PaO_2_ | -0.206 | **0.004** |
| FEV_1_ percent predicted | -0.005 | 0.94 |
| KCO percent predicted | -0.237 | **0.003** |
| Age at recruitment | 0.073 | 0.31 |
| BMI | -0.036 | 0.63 |
| *EDN1* GG or (GT + TT) | 0.090 | 0.19 |

| **RVSP vs Predictors** | **Standardised Coefficients** | ***P* value** |
| --- | --- | --- |
| PaO_2_ | -0.206 | **0.004** |
| FEV_1_ percent predicted | 0.004 | 0.95 |
| KCO percent predicted | -0.202 | **0.01** |
| Age at recruitment | 0.032 | 0.66 |
| BMI | -0.018 | 0.81 |
| *NOS3*-VNTR (4aa +4ab) or 4bb | -0.216 | **0.002** |
| *VEGFA* GG or (GC+CC) | 0.062 | 0.36 |
| *NOS3*-298 (GG + GT) or TT | -0.011 | 0.88 |
| *SLC6A4* LL or(LS + SS) | 0.107 | 0.12 |
| *PTGIS* (CC + CA) or (AA) | -0.052 | 0.44 |
| *ACE* II or (ID + DD) | -0.056 | 0.41 |
| *EDN1* GG or (GT+TT) | 0.058 | 0.40 |

| **RVSP vs Predictors** | **Standardised Coefficients** | ***P* value** |
| --- | --- | --- |
| FEV_1_ percent predicted | -0.082 | 0.20 |
| KCO percent predicted | -0.249 | **0.0004** |
| Age at recruitment | 0.126 | **0.043** |
| BMI | 0.019 | 0.78 |
| *NOS3*-VNTR (4aa +4ab) or 4bb | -0.162 | **0.009** |
| *VEGFA* GG or (GC+CC) | 0.111 | 0.07 |
| *NOS3*-298 (GG + GT) or TT | 0.011 | 0.86 |
| *SLC6A4* LL or(LS + SS) | 0.101 | 0.10 |
| *PTGIS* (CC + CA) or (AA) | -0.005 | 0.93 |
| *ACE* II or (ID + DD) | -0.047 | 0.43 |
| *EDN1* GG or (GT+TT) | 0.068 | 0.27 |

S3: Association of genotypes with RVSP measurements, using *t*-test

| **Genotype Group** | **Number** | **Mean** | **Standard Deviation** | **Mean Difference**  **(95% CI)** | ***P* value** |
| --- | --- | --- | --- | --- | --- |
| *ACE* II or ID | 208 | 44.5 | 13.1 | 0.91  (-2.58 to 4.39) | 0.61 |
| *ACE* DD | 69 | 43.6 | 11.6 |  |  |
| *ACE* II | 69 | 45.6 | 14.2 | 1.72  (-1.76 to 5.20) | 0.33 |
| *ACE* ID or DD | 208 | 43.9 | 12.2 |  |  |
| *VEGFA* GG or GC | 240 | 44.0 | 12.8 | -2.01  (-6.38 to 2.37) | 0.39 |
| *VEGFA* CC | 38 | 46.0 | 12.1 |  |  |
| *VEGFA* GG | 123 | 42.8 | 12.0 | -2.55  (-5.56 to 0.47) | 0.10 |
| *VEGFA* GC or CC | 155 | 45.4 | 13.2 |  |  |
| *SLC6A4* LL or LS | 221 | 43.9 | 13.0 | -1.12  (-4.93 to 2.70) | 0.57 |
| *SLC6A4* SS | 53 | 45.1 | 11.3 |  |  |
| *SLC6A4* LL | 90 | 42.7 | 12.6 | -2.10  (-5.30 to 1.09) | 0.20 |
| *SLC6A4* LS or SS | 184 | 44.8 | 12.6 |  |  |
| *NOS3*-298 GG or GT | 255 | 44.2 | 12.5 | -1.48  (-7.06 to 4.10) | 0.60 |
| *NOS3*-298 TT | 22 | 45.6 | 15.7 |  |  |
| *NOS3*-298 GG | 120 | 44.4 | 12.6 | 0.30  (-2.75 to 3.34) | 0.85 |
| *NOS3*-298 GT or TT | 157 | 44.1 | 12.9 |  |  |
| *NOS3*-VNTR aa or ab# | 78 | 47.2 | 14.5 | 4.01  (0.69 to 7.33) | **0.018** |
| *NOS3*-VNTR bb | 199 | 43.2 | 11.9 |  |  |
| *PTGIS* CC or CA | 262 | 44.3 | 12.7 | 0.05  (-6.71 to 6.62) | 0.99 |
| *PTGIS* AA | 15 | 44.3 | 13.8 |  |  |
| *PTGIS* CC | 172 | 43.9 | 12.2 | -0.93  (-4.04 to 2.18) | 0.56 |
| *PTGIS* CA or AA | 105 | 44.9 | 13.6 |  |  |
| *EDN1* GG or GT | 242 | 44.4 | 12.9 | 3.66  (-1.66 to 8.97) | 0.18 |
| *EDN1* TT | 24 | 40.7 | 9.3 |  |  |
| *EDN1* GG | 159 | 43.5 | 12.8 | -1.26  (-4.37 to 1.85) | 0.43 |
| *EDN1* GT or TT | 107 | 44.8 | 12.3 |  |  |

# The *NOS3* VNTR 4aa genotypes were present in only 8 subjects, and this number was too small to meaningfully compare in the t-test analyses.

S4: Association of genotypes with FEV_1_ percent predicted, using *t-*test

| **Genotype Group** | **Number** | **Mean** | **Standard Deviation** | **Mean Difference**  **(95% CI)** | ***P* value** |
| --- | --- | --- | --- | --- | --- |
| *ACE* II or ID | 425 | 49.1 | 21.5 | -4.50  (-8.52 to -0.48) | **0.028** |
| *ACE* DD | 153 | 53.6 | 22.2 |  |  |
| *ACE* II | 142 | 49.6 | 20.9 | -0.88  (-5.01 to 3.26) | 0.68 |
| *ACE* ID or DD | 436 | 50.5 | 22.1 |  |  |
| *VEGFA* GG or GC | 502 | 49.7 | 21.9 | -3.83  (-9.11 to 1.45) | 0.16 |
| *VEGFA* CC | 75 | 53.5 | 20.4 |  |  |
| *VEGFA* GG | 246 | 48.7 | 21.0 | -2.55  (-6.14 to 1.04) | 0.16 |
| *VEGFA* GC or CC | 331 | 51.3 | 22.2 |  |  |
| *SLC6A4* LL or LS | 464 | 50.0 | 21.7 | -0.156  (-4.75 to 4.44) | 0.95 |
| *SLC6A4* SS | 107 | 50.2 | 22.1 |  |  |
| *SLC6A4* LL | 179 | 49.7 | 22.7 | -0.519  (-4.38 to 3.34) | 0.79 |
| *SLC6A4* LS or SS | 392 | 50.2 | 21.4 |  |  |
| *NOS3*-298 GG or GT | 534 | 50.5 | 22.0 | 3.12  (-3.79 to 10.04) | 0.36 |
| *NOS3*-298 TT | 41 | 47.4 | 18.3 |  |  |
| *NOS3*-298 GG | 258 | 50.1 | 21.0 | -0.393  (-3.97 to 3.19) | 0.83 |
| *NOS3*-298 GT or TT | 317 | 50.5 | 22.3 |  |  |
| *NOS3*-VNTR aa or ab | 162 | 50.7 | 20.4 | 0.844  (-3.11 to 4.79) | 0.68 |
| *NOS3*-VNTR bb | 413 | 49.9 | 22.2 |  |  |
| *PTGIS* CC or CA | 536 | 50.0 | 21.6 | -2.38  (-9.22 to 4.46) | 0.50 |
| *PTGIS* AA | 42 | 52.4 | 23.1 |  |  |
| *PTGIS* CC | 336 | 50.0 | 21.0 | -0.248  (-3.85 to 3.35) | 0.89 |
| *PTGIS* CA or AA | 242 | 50.3 | 22.8 |  |  |
| *EDN1* GG or GT | 500 | 50.2 | 22.0 | 2.33  (-4.54 to 9.20) | 0.51 |
| *EDN1* TT | 42 | 47.9 | 19.1 |  |  |
| *EDN1* GG | 322 | 50.0 | 22.4 | -0.054  (-3.80 to 3.69) | 0.98 |
| *EDN1* GT or TT | 220 | 50.0 | 20.9 |  |  |

S5: Association of genotypes vs KCO percent predicted, using *t*-test

| **Genotype Group** | **Number** | **Mean** | **Standard Deviation** | **Mean Difference (95% CI)** | ***P* value** |
| --- | --- | --- | --- | --- | --- |
| *ACE* II or ID | 49 | 58.3 | 23.1 | 0.666  (-3.57 to 4.90) | 0.76 |
| *ACE* DD | 150 | 57.6 | 21.2 |  |  |
| *ACE* II | 136 | 60.3 | 24.9 | 2.93  (-1.44 to 7.30) | 0.19 |
| *ACE* ID or DD | 423 | 57.4 | 21.8 |  |  |
| *VEGFA* GG or GC | 485 | 58.0 | 22.4 | -1.10  (-6.68 to 4.47) | 0.70 |
| *VEGFA* CC | 73 | 59.1 | 23.9 |  |  |
| *VEGFA* GG | 241 | 58.4 | 23.0 | 0.34  (-3.46 to 4.13) | 0.86 |
| *VEGFA* GC or CC | 317 | 58.0 | 22.3 |  |  |
| *SLC6A4* LL or LS | 451 | 58.4 | 22.6 | 1.19  (-3.67 to 6.06) | 0.63 |
| *SLC6A4* SS | 101 | 57.2 | 22.0 |  |  |
| *SLC6A4* LL | 176 | 58.0 | 22.4 | -0.326  (-4.36 to 3.71) | 0.87 |
| *SLC6A4* LS or SS | 376 | 58.3 | 22.5 |  |  |
| *NOS3*-298 GG or GT | 515 | 58.7 | 22.8 | 7.86  (0.71 to 15.01) | **0.031** |
| *NOS3*-298 TT | 41 | 50.9 | 16.7 |  |  |
| *NOS3*-298 GG | 250 | 58.5 | 24.0 | 0.605  (-3.17 to 4.38) | 0.75 |
| *NOS3*-298 GT or TT | 306 | 57.9 | 21.3 |  |  |
| *NOS3*-VNTR aa or ab | 156 | 58.2 | 21.9 | 0.156  (-4.04 to 4.35) | 0.94 |
| *NOS3*-VNTR bb | 400 | 58.1 | 22.9 |  |  |
| *PTGIS* CC or CA | 519 | 57.6 | 22.3 | -6.71  (-13.97 to 0.56) | 0.07 |
| *PTGIS* AA | 40 | 64.3 | 25.0 |  |  |
| *PTGIS* CC | 327 | 58.5 | 21.4 | 0.87  (-2.95 to 4.68) | 0.66 |
| *PTGIS* CA or AA | 232 | 57.6 | 24.2 |  |  |
| *EDN1* GG or GT | 483 | 57.5 | 22.5 | -7.07  (-14.38 to 0.25) | 0.06 |
| *EDN1* TT | 40 | 64.6 | 24.5 |  |  |
| *EDN1* GG | 310 | 57.5 | 22.4 | -1.31  (-5.28 to 2.66) | 0.52 |
| *EDN1* GT or TT | 213 | 58.8 | 23.1 |  |  |

**References**

1. Cheon KT, Choi KH, Lee HB, Park SK, Rhee YK, Lee YC: **Gene polymorphisms of endothelial nitric oxide synthase and angiotensin-converting enzyme in patients with lung cancer**. *Lung* 2000, **178**(6):351-360.

2. Novoradovsky A, Brantly ML, Waclawiw MA, Chaudhary PP, Ihara H, Qi L, Eissa NT, Barnes PM, Gabriele KM, Ehrmantraut ME *et al*: **Endothelial nitric oxide synthase as a potential susceptibility gene in the pathogenesis of emphysema in alpha1-antitrypsin deficiency**. *Am J Respir Cell Mol Biol* 1999, **20**(3):441-447.

3. Gao PS, Kawada H, Kasamatsu T, Mao XQ, Roberts MH, Miyamoto Y, Yoshimura M, Saitoh Y, Yasue H, Nakao K *et al*: **Variants of NOS1, NOS2, and NOS3 genes in asthmatics**. *Biochem Biophys Res Commun* 2000, **267**(3):761-763.

4. Nakayama T, Soma M, Saito S, Honye J, Yajima J, Rahmutula D, Kaneko Y, Sato M, Uwabo J, Aoi N *et al*: **Association of a novel single nucleotide polymorphism of the prostacyclin synthase gene with myocardial infarction**. *Am Heart J* 2002, **143**(5):797-801.

5. Eddahibi S, Humbert M, Fadel E, Raffestin B, Darmon M, Capron F, Simonneau G, Dartevelle P, Hamon M, Adnot S: **Serotonin transporter overexpression is responsible for pulmonary artery smooth muscle hyperplasia in primary pulmonary hypertension**. *J Clin Invest* 2001, **108**(8):1141-1150.

6. Watson CJ, Webb NJ, Bottomley MJ, Brenchley PE: **Identification of polymorphisms within the vascular endothelial growth factor (VEGF) gene: correlation with variation in VEGF protein production**. *Cytokine* 2000, **12**(8):1232-1235.
